# Mothers’ perception of childhood injuries, child supervision and care practices for children 0–5 years in a peri-urban area in Central Uganda; implications for prevention of childhood injuries

**DOI:** 10.1186/s40621-019-0211-1

**Published:** 2019-08-01

**Authors:** Godfrey Siu, Anthony Batte, Brenda Tibingana, Kennedy Otwombe, Richard Sekiwunga, Nino Paichadze

**Affiliations:** 1 Child Health and Development Centre, College of Health Sciences, Makerere University Kampala, Kampala, Uganda; 2 Department of Anesthesia and Perioperative Medicine, Centre for Medical Evidence, Decision Integrity and Clinical Impact (MEDICI), University of Western Ontario, London, ON Canada; 3 Statistics and Data Management Centre, Peri-natal HIV Research Unit, Faculty of Health Sciences, University of the Witwatersrand, Johannesburg, South Africa; 4 Department of Global Health, Milken Institute School of Public Health, George Washington University, Washington, DC USA

**Keywords:** Childhood injuries, Injuries, Child supervision, Uganda

## Abstract

**Background:**

Injuries are a major concern in childhood. They are especially associated with high morbidity, disability and death in low-income countries. This study aimed at describing mothers’ perceptions, child supervision and care practices for children 0–5 years old and how these influence prevention of childhood injuries among children in peri-urban areas of Wakiso district, Uganda.

**Methods:**

In this qualitative study, 10 in-depth interviews and 4 focus group discussions were held with mothers of children aged 0–5 years living in peri-urban areas of Wakiso district, Uganda. The interviews were audio recorded in the local language (Luganda). The audios were transcribed verbatim and later translated into English. We conducted thematic analysis for transcripts from the focus group discussions and in-depth interviews.

**Results:**

Most respondents considered injuries as inevitable events among children, although, they acknowledged the impact of injuries on children’s health. Close child supervision was highlighted as key in preventing injuries. Hostile situations that place children at increased risk of injuries in this setting include: lack of adult supervision, harsh punishments and lack of safe play areas.

**Conclusion:**

Our study highlights the different aspects of child care in low resource settings which put children at an increased risk of injuries. Injury prevention programs for children living in low resource settings should thus be aimed towards improving caregivers’ perceptions towards injuries, child supervision, care practices and the children play environment.

## Background

Childhood injuries are a serious problem of public health concern (WHO 2008). They are among the leading causes of childhood deaths and disability in low- and middle-income countries (LMICs) where at least 90% of such deaths and disabilities occur (Harvey, Towner, Peden, Soori, & Bartolomeos, 2009; Khan et al., 2015). Sub-Saharan Africa, suffers the greatest burden of childhood injuries, including falls, road traffic injuries, and burns (Bartlett, 2002). It is therefore important that injury prevention initiatives are enhanced in Sub-Saharan Africa to improve child health.

In Uganda, previous research shows a high incidence of unintentional childhood injuries in the urban areas, and that child injuries constitute a significant proportion of emergency unit visits and hospital admissions among children (Hsia et al., 2011; Mutto, Lawoko, Nansamba, Ovuga, & Svanstrom, 2011; Nakitto, Mutto, Howard, & Lett, 2008). However, most of these studies are quantitative in design, are largely concerned with describing the epidemiology of childhood injuries, and have mostly been conducted in Kampala city and within a hospital or school setting. Limited research has been conducted in peri-urban areas or rural areas of Uganda, in spite of the fact that the rapid unplanned urbanization in LMICs continues to produce squatter camps, slums and informal urban settlements which pose high risks of childhood injury (Harvey et al., 2009). There is limited qualitative research with primary caregivers exploring their perception of unintentional childhood injuries and care practices. In the current qualitative study, we aim to add-on to the existing literature by discussing mothers’ perceptions of childhood injuries, their child care practices, and the perceived need for interventions to reduce child injuries in peri-urban areas of Uganda.

## Methods

### Study setting

We collected both survey and qualitative data in Kigungu and Bweya parishes in Wakiso district, about 40 kms from Kampala, between August–September 2015. Results of the survey are reported elsewhere (Batte et al., 2018). Kigungu parish is located in Entebbe municipality division B, and the parish has 1,175 households with a total population of 4,526 people (adults and children) (UBOS, 2005). Bweya parish is located in Ssisa subcounty and has 1,435 households and a total population of 6,217 people (adults and children). These parishes have populations of mixed ethnicity, the majority being the *Baganda.* The parishes have peri-urban characteristics, with a variety of occupations, including fishing, trading, subsistence farming and formal employment.

### Data collection

We conducted a total of 10 in-depth interviews with mothers (or female primary caregivers in absence of a biological mother) of children aged 0–5 years – mothers/caretakers aged between 20 years and 45 years, and four focus group discussions (FGDs) of 6–8 participants were conducted with mothers/caretakers. We selected mothers who had not previously participated in the quantitative study (Batte et al., 2018). The interviews and focus group discussions were led by a male researcher with a Bachelor’s degree in social sciences and a Master’s degree in population and reproductive health. He was assisted by a female research assistant with a Bachelor’s degree in population studies. Both had previous experience of conducting qualitative studies, were fluent in Luganda, the local language of the community, and had no pre-existing relationship with the community. The interviews were conducted at the respondent’s home, while focus groups were conducted within the community, usually at school within the locality. The interviews and FGDs elicited data on a range of topics including the types of injuries common among children aged 0–5 years, caregivers’ perception of importance and severity of different childhood injuries, risk factors, parenting practices, and challenges keeping children safe. The data were audio recorded in Luganda, a widely spoken local language and back-up hand written notes were taken.

### Analysis

The recordings of the interviews and focus group discussions were transcribed and translated into English by the research assistants, and checked for completeness and accuracy by the primary authors. We then conducted thematic analysis manually (Creswell & Poth, 2017). The transcripts were read and a preliminary code book developed following the interview guide, and a debriefing with the research assistants was done to confirm the key themes. Five main themes were identified, including; importance of childhood injuries, environment and risk, child supervision and care practices, and impact of injuries on children. Data were then coded using a matrix table in Microsoft Excel. Focus group discussions were summarised against themes that emerged from the in depth interviews. Finally the manuscript was drafted and reviewed by all the authors.

## Results

### Demographic characteristics

All mothers who were interviewed had only attained either primary level education or secondary level education and none of them had tertiary level education. All mothers except interviewee 2 were biological mothers to the children, and reported that they were married (Table 1).

**Table 1:** Demographic characteristics of mothers interviewed

Interviewee	Parish	Age	Marital status	Education level	Number of children below age 5
1	Kigungu	23 yrs	Married	Primary	2
2	Kigungu	45 yrs	Widow	Primary	2
3	Kigungu	24 yrs	Married	Primary	2
4	Kigungu	34 yrs	Married	Primary	3
5	Kigungu	25 yrs	Married	Primary	2
6	Bweya	30 yrs	Married	Secondary	3
7	Bweya	27 yrs	Married	Secondary	1
8	Bweya	23 yrs	Married	Primary	1
9	Bweya	24 yrs	Married	Secondary	1
10	Bweya	26 yrs	Married	Primary	3

Regarding focus groups, a total of 27 participants were involved, and as with the interviews, they had all attained either primary level education or secondary level education (Table 2).

**Table 2:** Focus group discussion participants

FGD group	Parish	Number of participants	Age range	Mar status			Education level
				Married	Single	Widow	
1	Kigungu	6	20–35	06	–	–	Primary
2	Bweya	7	25–35	05	02	–	Primary to Secondary
3	Bweya	6	25–40	04	01	01	Primary to secondary
4	Kigungu	8	20–35	07	01	–	Primary

### The importance parents attach to childhood injuries

Caregivers from both the in-depth interviews and focus group discussions reported that their child had ever suffered some form of unintentional injury. Most parents believed that unintentional injuries are inevitable events that could not be avoided as children grow up, and as such, it is not a matter of whether a child will get injured but rather when the child will get injured. One mother described thus: *[…] like getting a wound, cutting herself, knocking her legs, falling down because they keep playing and they (injuries) are unavoidable.* However, other mothers suggested that the risk of children getting injured was greater and unavoidable in certain contexts, especially away from home, in particular implying that absence of parental care made injuries more likely to happen. A mother discussed this issue as follows: *Now like if this child turns four years and starts school, she may get a problem (an injury) as you’re not near her and yet if she was near, you never know she could not have got it.*

To explore the relative importance of child injuries compared to other child care concerns among mothers, we asked mothers at the beginning of the interview and focus group discussions, to first freely describe the greatest fears they had in their day-to-day care-giving for young children aged below 5 years. Mothers raised a variety of issues ranging from poverty to lack of food and school fees, infections/falling sick/health, getting lost/stolen, child sacrifice, and a range of injuries – burns, accidents, drowning, dog bites, and playing on the roads. For instance a mother aged 24 years expressed her concerns: *I fear for him getting burnt, or for him getting knocked … but even to steal him away from me. That is what I fear most.* A mother in her 40’s said: *The things I fear particularly are her studies …*. *Then also her medication because as I have told you I take care of the children myself and I have to look after their welfare.* The spontaneous inclusion of a list of injuries by mothers amongst their greatest fears regarding childcare, during the interviews and group discussions suggests that; in this study area, child injuries are indeed an important issue of everyday concern.

When we further probed for the importance of the different forms of child injuries, we noted that mothers attached varying significance to child injuries depending on the cause and age of the child. A young mother vividly recalled a fatal child injury that occurred in her neighborhood and anxiously reported: *there is a lady whose child got burnt and died. They took her to grade B (in reference to Entebbe Hospital) and also up to Mulago hospital (Uganda National Referral hospital) but the child died. It was hot water in the kettle that the child knocked and it poured on the stomach injuring the child.* Similarly, another young mother aged 24, explained: *Ok …*. *the severe ones are if the child gets burnt by fire and also if the friend has stoned him may be in the eye and he gets hurt... That is also severe.* However, the same felt that simple cuts were not a major concern and could be left untreated.

Mothers from both the focus group discussions and in-depth interviews reported a wide range of child injuries. The most common injuries reported include burns, bruising and fractures. A mother aged 25 years described thus: *They get burnt and others fall in the swamps but thereafter removed, even climbing trees and they fall down. They also fight with their friends for example, this young one of mine has many scars but it is the friends who burnt him, they even light the fire when you’re not around and one time they lit a basin and they burnt his hands.*

### Mothers’ perception of children’s immediate environment and risk of injuries

Most mothers believed that the immediate environment (home, neighborhood, and school) in which their children lived was largely unsafe, posing a risk to injuries. Many examples of risks cited by mothers suggest that there was far greater level of risk of a child getting injured in the neighborhood/outdoors than when a child was indoors. Among the many factors mothers identified were the busy roads and careless motorists in their areas, presence of many open pits and swamps especially in Bweya parish, and the presence of fruit trees in some homes and neighborhoods which children are tempted to climb. Others are; the frequent burning of garbage in open areas near homesteads and household fires, and a host of other risks at school. Several mothers were concerned that there were many abandoned and/or active sand and stone mining pits, as well as construction pits dug in the rapidly developing Bweya, that posed danger for their children. Some mothers cited tragic incidences in which children or adults have fallen in those pits or swamps and had to be rescued.

Child related risk factors identified by the mothers included: child not willing to listen to the parental instructions, for example, against climbing trees; conflict and fights among children, and child’s ignorance of the risk entailed in their activity, as a mother aged 30 reported: *Like this size (at this age) they may be playing with friends and they keep stoning each other, pushing him on the ground, he may be running and he falls down and breaks badly.* Participants reported that younger children may throw stones at each other as part of play, while for older ones in some instances, stones are used as a weapon during fights, risking causing severe injuries to others. Children getting stoned by fellow children is an interesting observation in this qualitative study, and is one of the factors less frequently reported in qualitative studies.

### Mothers’ child supervision and care practices

Although many mothers often reported about other mothers’ care practices and child supervision, it may be inferred from some of their responses and examples that those experiences largely applied to themselves as well and exposed their children to risk of injuries. Many of the care practices by mothers were positive and aimed at minimizing the risk of their children being injured although some of the mothers’ care practices inadvertently caused injury or posed a danger to their children.

#### Personal responsibility in child supervision

Mothers reported that they themselves had the primary responsibility to look after children, and hence personally supervised the children. However, the issue of balancing child care with household chores and work away from home, was one of the most commonly discussed child supervision challenge. The greatest obstacle to proper child supervision resulted from mothers, for the most part, doing the chronic household chores and the resulting exhaustion due to heavy chores, or for the few employed ones, being away ‘busy at the work place’. Spousal conflict was also identified as a major factor that undermined mothers’ ability to provide appropriate care and supervision of their children. Although this issue was identified by very few mothers, the respondents who discussed it were emphatic about it, suggesting that this was an important issue. One of the mothers reported: *You know everyone has personal problems, there are those who don’t care because the father of the child abandoned her, and that she does not have help especially when the child falls sick so the woman may lose hope.*

Maternal alcohol abuse and frustration of some mothers following lack of spousal support impacted the way the mothers supervised their children. The phrase ‘I don’t care attitude’ was sometimes used to describe the care practices of these mothers. This was attributed mostly to mothers who lived alone and were therefore frustrated caregivers or those who were abusing alcohol. A mother said: *There are women who drink alcohol so sometimes she may sleep off and the child moves out to touch hot fire and gets burnt.*

#### Overreliance on child’s siblings

Furthermore, other mothers reported being over reliant on older siblings, but who themselves were found to be children, to care for the young ones. Other mothers often allowed the younger children to join play with older children in the neighborhood. This child care practice was a major care practice that left little children vulnerable to sustaining injuries in their everyday life, as discussed in an FGD: *Imagine the mother is an alcoholic. The father has gone to work. Now it is up to the older child to look after his siblings. For example, my [polygamous] husband’s daughter, she has been looking after her siblings when her mother is not around, she has to make the fires and the baby is crying. An accident can happen. She can be burnt or even cut herself.*

#### Harsh discipline to deter children from risky situations

Mothers reported using harsh discipline to deter children from risky places and to enforce appropriate conduct. However, in the process of exercising “strict discipline to protect children”, these measures inadvertently cause injury to the children, as some mothers admitted. For example, a mother in her 20’s revealed: *A child may, for example, abuse me and I throw a stone at him or her but accidently it hits the head causing injury which was as a result of being stubborn.*

Similarly, another mother reported: *Some parents get angry very fast and if a child takes some money for use they beat them so much. Some of them if a child eats the mother’s food, they beat them seriously, also if they send the child to the well and they delay, they are beaten.* Reporting on severe forms of punishment used by mothers, another participant described circumstances when some mothers burn their children for wrong doing, saying: *[…*] *and also some parents burn their children. … It may be due to something small especially if they are not the biological mothers.*

#### ‘Tight marking’ and keeping children at arm’s length

Mothers’ care practices could be described as ‘tight marking the children,’ as they usually “*kept children within their reach”* as they went about their chores. Several mothers described the importance of paying keen attention to one’s children, and keeping them at an arms-length, especially for the much younger children. One mother said: *One should always move with the child. For my case I work near the road but most times I carry the child on my back as I work [Mother of two, age 23].*

Mothers reported that they are always suspicious of the environment and did not underestimate the risks to their children. For example, one mother who assessed the swampy areas to be too risky for her children articulated: *I don’t go to the well (spring) with the young ones, so I lie to them that there are snakes or dogs in the well and they fear going there [Mother of three, age 26].*

### Awareness of impact of injuries on children

All mothers understood the dangers of injuries on their children. Mothers reported that injuries could lead to death, wounds, fractures, lifelong scars, and that it could even affect school performance. Mothers’ accounts provided insights into their perceptions of the different injuries and their relative severity and implication. We found that mothers perceived injuries associated with drowning as most severe, and those associated with fighting with friends as least severe. For example, when a mother was asked which injury type worried her the most, she stated that it was *‘ … to fall in the swamp … Because if he doesn’t have any one to help him [out] then he will die.* However, this same mother when asked which injury does not come across as severe to her, she stated that it was *‘fighting with their friends...[because] when they fight they don’t hurt each other so much.’* Another mother identified *‘falling in a pit, and falling from the tree as most worrisome, […]* *because he may fall in a deep pit and breaks the chest then it becomes difficult to heal.* These perceptions were similar to those shared by the participants in FGDs: *There are some injuries that are not severe like a child getting a razor blade or knife and cuts himself. But when it is burns, there you take the child straight to hospital.*

Mothers’ perception of severity of an injury seemed to vary by their age, with the younger mothers especially appearing more sensitive and fearful than the older mothers. Older mothers appeared less bothered with child injuries probably because they have more experience dealing with these injuries.

## Discussion

We conducted a qualitative study to explore perception of childhood injuries, child supervision and care practices for children 0–5 years. A number of themes have emerged from this study providing insights on the importance parents attach to childhood injuries, the mothers’ perception of risk factors for childhood injuries, child supervision and care practices, and the mothers’ awareness of the impact of injuries on children. Our study had some limitations, suggesting the need to interpret these findings in context. First, we conducted the study with only mothers. It would have been more insightful to include fathers, as well as other caregivers since care giving in this context is socially distributed and not restricted to mothers alone. Second, information concerning intentional injuries usually generates embarrassment and feelings of anger among respondents and may thus be underreported (Scheidt, Brenner, Rossi, Clyman, & Boyle, 2000). Nevertheless, we heard many important insights from mothers, who are often the primary caregivers for this category of children. In addition, these qualitative data complement the quantitative data from the same population described elsewhere (Batte et al., 2018).

The study found that mothers consider unintentional injuries as an inevitable occurrence in the child’s lifetime. The perception that injuries are inevitable is a significant hindrance for caretakers’ involvement in the various injury prevention programs (Ablewhite et al., 2015). This necessitates integration of behaviour and perception change intervention models in any programs aimed at control of childhood injuries (Gielen & Sleet, 2003).

Although mothers report that injuries are inevitable occurrences in children, they displayed awareness and expressed fear that injuries could impact the health of their children in the long-term, including possibility of death. The importance attributed to childhood injuries, in particular the awareness of the potential severity of some types of injuries, including permanent disability and death, in this qualitative study confirms the findings we described in a survey of mothers in this setting (Batte et al., 2018), and has been reported in other quantiative studies in Uganda and other low and middle income countries (Kobusingye, Guwatudde, & Lett, 2001). This concern and fear might be harnessed by injury prevention programs to drive positive behaviour of the mothers to get involved in prevention of childhood injuries.

Mothers identified key environment and child risk factors for injuries, including the unsafe environment and the unsafe play practices by the children. Environmental risks have been identified in other studies as well (Munro, van Niekerk, & Seedat, 2006), and the contribution of children’s behaviour and age towards the risk for injuries has also been described elsewhere (Pant et al., 2015). However, in our study the attribution of injury risk factors to external factors other than the individual mothers could be an expression of a strong external locus of control (Rotter, 1966). In the context of this theory, individuals with strong external locus of control tend to attribute occurrences on factors outside their control; attributing occurrences to environment, chance and fate instead of their individual attributes and skills which would positively influence behaviour to foster change (Rotter, 1966). Studies have demonstrated that parents who attribute occurrence of childhood injuries to external factors such as fate and luck, have an increased incidence of injuries among their children (Morrongiello & House, 2004). From this study it could be seen that mothers perceived injuries as inevitable and their attribution of the occurrence of injuries to unsafe environment needs to be further explored since this could be an expression of external locus of control among these mothers. It is important that further studies are conducted to assess the interplay of all these various factors and attributes in occurrence of childhood injuries. This qualitative data could not offer sufficient insight into the specific patterns of the association between perceptions of childhood injuries and mothers’ age or number of children under her care, but quantitative data from the same population suggests that injuries occur less frequently among children under the care of older mothers (Batte et al., 2018).

Inadequate supervision of children was identified as a common negative practice by mothers. This has been reported in various studies as a driver for increased incidence of injuries in children (Landen, Bauer, & Kohn, 2003; Morrongiello, Corbett, McCourt, & Johnston, 2006; Schnitzer, Dowd, Kruse, & Morrongiello, 2014). Although most mothers in this study were the ‘stay home mothers’, the chronic nature of domestic chores they are involved in during the day greatly affected the time available for child care and supervision. This threatens to erode the advantage of such mothers being around the children. There is need to investigate further how mothers prioritize conflicting domestic obligations, and develop educational and basic technology based interventions to support them to effectively manage child supervision amidst other priorities. Other negative practices which were identified to be increasing the childhood injury risks included negligence, spousal conflict and drunkenness which compromised the care mothers offered to the children. Injuries inflicted on children as punishment were also highlighted by mothers in this study as a practice which causes injuries in the children. The use of punishment which is justified as disciplining the child and a component of child rearing is widely recognised as a negative child care practice associated with occurrence of injuries in children (Akmatov, 2011; Mudany, Nduati, Mboori-Ngacha, & Rutherford, 2013). However, some mothers expressed good child care practices including close supervision of the children and protecting children from accessing risky environments.

## Conclusions and recommendations

Mothers perceive injuries as important occurrences which result in morbidity, disability and even death of children. This awareness may be harnessed by injury control programs to motivate mothers in embracing injury prevention interventions in these low income settings. Key issues that need to be targeted in prevention of childhood injuries include unsafe play environments, inadequate child supervision and poor child care practices. However, the perception that injuries are inevitable occurrences in the course of raising children is an extremely important barrier that could significantly undermine any prevention efforts. Addressing this attitude among mothers should be an important component of any childhood injuries prevention program.

## Data Availability

The datasets used and/or analysed during the current study are available from the corresponding author on reasonable request.

## Abbreviations

FGD: Focus group discussion
LMICs: Low- and middle-income countries
